# Sarcopenia and Sarcopenic Obesity and Osteoarthritis: A Discussion among Muscles, Fat, Bones, and Aging

**DOI:** 10.3390/life13061242

**Published:** 2023-05-24

**Authors:** Maria Spanoudaki, Constantinos Giaginis, Maria Mentzelou, Alexia Bisbinas, Evangelos Solovos, Konstantinos Papadopoulos, Ioannis Paliokas, Christiana Zidrou, Antonis Cheimaras, Maria Hassapidou, Athanasios N. Papadopoulos, Sousana K. Papadopoulou

**Affiliations:** 1 Department of Nutritional Sciences and Dietetics, School of Health Sciences, International Hellenic University, 57400 Thessaloniki, Greece; 2 Clinical Dietetics & Nutrition Department, 424 General Military Hospital, New Efkarpia Ring Road, 56429 Thessaloniki, Greece; 3 Department of Food Science and Nutrition, School of Environment, University of Aegean, 81400 Myrina, Greece; 4 AHEPA Hospital, 54636 Thessaloniki, Greece; 5 A Orthopaedic Clinic, 424 General Military Hospital, 56429 Thessaloniki, Greece; 6 School of Economics and Business Administration, International Hellenic University, 57001 Thessaloniki, Greece; 7 G. Papageorgiou Hospital, 56429 Thessaloniki, Greece

**Keywords:** osteoarthritis, sarcopenia, sarcopenic obesity, middle-aged adults, older adults, functionality

## Abstract

Aging is a physical procedure for people and nature. Our aging world is expanding because of the life span extension. Aging has a crucial relationship with our body composition (muscles, bones, and adipose tissue), which is characterized by an increase in fat mass and a gradual decrease in muscle mass and strength and bone density. These alterations affect physical performance and impact quality of life enhancing the risk for non-communicable diseases, immobilization, and disability. As far we know, osteoarthritis of lower limbs, sarcopenic obesity, and muscle mass and/or strength loss are treated separately. However, bones, muscles, adipose tissue, and aging appear to have an interconnection through a dialogue as they talk to each other. Health disorders are coming into the surface when this relationship is disrupted. The aim of our study is to search deeper into this interconnection, so that when adipose tissue increases, we have to take a look into the condition of muscle mass, bone, and connective tissue and vice versa, through the assessment of physical performance. Consequently, the triad muscle-bone-adipose tissue disorders by aging should be treated as a single entity.

## 1. Introduction

Our world ageing pattern has been altered with adults aged over 65 being the fastest growing age group, counting for 13% of the global population and expected to reach around 2 billion by the year 2050. The expansion of life expectancy at age 65 is expected to have a significant upward trend during the five-year period 2045–2050, resulting in a dramatic increase in the number of people aged over 80 in the coming decades [1,2]. Continuous progress in science and technology has resulted in greater life expectancy. Thus, in our aging world, sarcopenia, osteoarthritis (OA), and obesity are becoming main health disorders, placing a great burden on the healthcare system.

A growing body of literature has focused on sarcopenic obesity (SO), introducing a new phenotype of obesity in the last decade. A decline in both muscle and bone mass and an increase in adipose tissue are observed due to biochemical changes and genetic and lifestyle factors [2,3]. Excessive body fat, independent of body weight, takes over key structures, such as muscle tissue and internal organs, leading to atrophy and dysfunction. On the other hand, sarcopenic osteoarthritis seems to be a novel term describing the co-existence of sarcopenia and osteoarthritis. Both musculoskeletal conditions lead older adults to inactivity, frailty, poor quality of life, and death [4,5]. Additionally, the risk of lower limb osteoarthritis is higher in sarcopenic obese people [6]. This appears to indicate a strong metabolic involvement of obesity in osteoarthritis pathogenesis [6,7]. Sarcopenic osteoarthritis in an obesogenic body environment appears to be a more complex condition as little evidence highlights this field [8,9]. Evaluation of this triad is considered difficult and a single practice for clinical management has not been adopted due to the lack of global treatment guidelines. Management of these three health disorders as one pathological entity is focused on body fat loss and muscle mass or strength retention and gain, but official recommendations as a global routine strategy have not been specified. On the other hand, international guidelines for sarcopenia evaluation and management have been edited for healthcare practitioners by Dent et al. [1], though the age of the target population was over 65 years (Table 1).

Osteoarthritis, sarcopenia, and sarcopenic obesity, when coexisting, are still poorly detected and under-treated. Identification of any of those conditions should result in detection for sarcopenia/sarcopenic obesity when osteoarthritis is present and vice versa. The aim of this study is to review and to bring on surface the latest approaches to the interconnection among sarcopenia, osteoarthritis, and sarcopenic obesity, their cause-effect relationship, and their impact on physical performance and functioning.

## 2. Methods

A comprehensive search of relevant studies published in the last decade (from 2012 to 2023) was conducted in PubMed, Elsevier, Google Scholar, Science Direct. Research terms were sarcopenia, sarcopenic obesity, osteoarthritis, sarcopenic osteoarthritis, knee or/and hip osteoarthritis, physical performance, functionality, performance deficit, arthroplasty, older adults, and middle-aged adults. Inclusion criteria were clinical trials, Randomized Control Trials (RCTs), prospective studies, cross-sectional studies, and cohort studies. Exclusion criteria were studies involving animals, age of participants <50 years old, studies involving athletes and Knee or/and hip injuries, case studies, non-randomized studies, and surveys not written in English.

### 2.1. Aging Impact on Bone, Muscle, and Adipose Tissue

Ageing is strongly associated with the development of sarcopenia, predisposition of fat mass, osteoporosis, and osteoarthritis, although it is not fully understood how the relationship between these tissues is affected by ageing [16]. Research shows that the association between muscle size and muscle and bone strength changes significantly with age, suggesting a possible inequality between these tissues [17]. The essential role of fat tissue in skeletal and bone mass cannot be neglected because adipocytes, myoblasts, and osteoblasts are coming from the common mesenchymal stem cells. There are marked cellular and molecular changes in bone and muscle cells with age [18,19], which probably explain these findings. These changes have been described to include the loss of osteocytes in the bone matrix and a reduction in the proliferative capacity of osteoprogens in the periosteum, which weaken the reaction of bone to muscle contraction and normal mechanical stimuli [7,8]. Another actor that has a key role in energy balance and muscle and bone metabolism is age-dependent: leptin. The term leptin comes from the Greek word “leptos”, which means thin, and it is a plyotropic adipokine, having cytokine and hormonal action. It is mainly secreted by white adipose tissue. However, other tissues such as brown adipose tissue, stomach, brain, skeletal muscles, and ovaries also produce leptin [20]. It has been highlighted that leptin sensitivity decreases, with concomitant hyperleptinemia, during age-related weight gain and in obesity in people of all ages and with different etiologies [21].

Ageing affects the action of leptin on bone metabolism in several ways [22,23]. Central leptin resistance, which is known to increase with ageing, is probably attenuating the central hypothalamic-mediated impacts of leptin [24]. This might have a strongly significant effect on bone marrow adipogenesis and bone marrow fat deposition with age [22,25].

Leptin receptors are plentiful in skeletal muscle, as well as in bone stromal cells. Evidence suggests that leptin might play an essential role in cross-communication between muscle and bone tissue [24,25]

Bone marrow lipogenesis is related to low bone mass in humans. Leptin can decrease bone marrow lipogenesis centrally via its receptors in the hypothalamus and directly via its receptors in bone marrow stem cells [22,26].

Nevertheless, central leptin resistance may increase with age, and low levels of circulating leptin have been observed in elderly people with frailty [21,22]. Therefore, aging seems to significantly modify the leptin-mediated intercellular signaling between different organs and tissues.

Leptin levels are positively associated with a lifespan of 100 years, and the proportion of leptin to adiponectin has been also positively associated with muscle strength in the elderly. In addition, higher leptin levels are related to a lower risk of dementia in older adults. Therefore, leptin signaling and changes in leptin sensitivity with aging may contribute to age-related dysregulation of multiple organs and tissues [25].

Mechanical loading is a basic mechanism connecting both tissues with a pivotal promotional role of physical activity. Moreover, skeletal muscle secretion comprises several bone-affecting markers such as insulin-like growth factor-1 (IGF-1), interleukin-6 (IL-6), interleukin-15 (IL-15), basic fiboblast growth factor-2 (FGF-2), myostatin, osteoglycine (OGN), and osteoactivin. While studies on the possible effects of bone on muscle metabolism are limited, few osteokines have been discovered. Prostaglandin E2 (PGE2), which is released by osteocytes and sclerostin, secreted by both cell types osteocalcin (OCN) and IGF-1, and produced by osteoblasts, potentially affects skeletal muscle cells. Cartilage, muscle, and adipose tissue are also probably engaged in this control pathway and should not be ignored [17].

### 2.2. Sarcopenia

Sarcopenia was recognized as a disease in 2016 and coded by the International Classification of Diseases (ICD). The European Working Group on Sarcopenia in Older People (EWGSOP) defined sarcopenia as a syndrome characterized by progressive loss of skeletal muscle mass, strength, power, and performance associated with a risk of disability, poor quality of life, and death [27]. The EWGSOP has classified sarcopenia as primary, age-related, and secondary, which includes sarcopenia associated with lack of physical activity (after prolonged bed rest, reduced physical activity, and sedentary lifestyle), diseases (advanced organ failure, inflammatory diseases, malignant diseases, and endocrine abnormalities), and nutrition (macronutrient and micronutrient deficient diet, malabsorption, gastrointestinal disorders, and drug-induced anorexia). Low muscle strength without loss of muscle mass is defined as dynapenia. WGSOP classifies sarcopenia as “pro-sarcopenia”, “sarcopenia”, and “severe sarcopenia” [28].

The “pro-sarcopenia” stage is characterized by reduced muscle mass without decline of muscle strength or functionality. This stage can only be detected by techniques that measure muscle mass accurately and the involvement of standardized populations [29].

The “sarcopenia” stage is characterized by reduced muscle mass and reduced muscle strength or functionality, while “severe sarcopenia” is characterized by reduced muscle mass, strength, and functionality [30]. Behind the decline in functional capacity due to the reduction in skeletal muscle mass, there is a pattern of metabolic-hormonal disturbances that gradually leads to atrophy of type IIa muscle fibers, reduction of motor neurons, and alteration of muscle architecture. Increased intramuscular fat deposition, decreased growth hormone (GH) secretion, and chronic presence of elevated levels of pro-inflammatory cytokines such as interleukin-6 (IL-6), interleukin-1 (IL-1), adipokin, and leptin are associated not only with aging, inadequate protein synthesis, development of insulin resistance, and low levels of vitamin 25(OH)D, but also impaired fatty acid oxidation due to mitochondrial dysfunction and oxidative stress, which comprise a disturbed metabolic profile that may exacerbate crucial risk factors for osteoarthritis [11,12,13,14,31]. Isoprostanes (biomarkers of oxidative stress), associated with cardiovascular disease, were negatively correlated with functional capacity in adults over 60 years of age with frailty syndrome. Low walking speed was negatively associated with IL-6 (B = −0.025 m/s, 95% CI, 0.04, −0.01) and urinary isoprostane concentrations (B = −0.019, 95% CI −0.03, −0.008), [18].

Regarding pro-inflammatory cytokines, leptin administration in vitro elevates the expression of myogenic genes in primary myoblasts and administration in vivo enhances the microRNA expression implicated in myogenesis [25].

Furthermore, a significant positive association has been found between leptin levels and abdominal muscle area and a strong correlation has been found between leptin levels and muscle radio-density in overweight and obese adults of mean age of 64 years old. Particularly, leptin was associated with muscle radio-density positively and strongly after adjustment for all covariates (age, sex, race/ethnicity, smoking, dyslipidemia, hypertension, physical activity, sedentary behavior, and abdominal subcutaneous and visceral adipose tissue). A1-SD increase in total abdominal, stability, and locomotor muscle radiodensity was associated with 31%, 31%, and 18% lower adiponectin levels (*p*  <  0.01 for all) and with 8.1% higher leptin levels [31].

Therefore, adiponectin and leptin levels are possibly related to muscle mass and quality, while lower muscle mass and function lead older people to frailty.

### 2.3. Sarcopenic Obesity

Baumgartner (2000) was the first to propose the term sarcopenic obesity, defining it as sarcopenia accompanied by an increase in adipose tissue. The current definition of sarcopenic obesity combines sarcopenia with concomitant obesity. Sarcopenia and obesity appear to have similar pathophysiological mechanisms and analogous causative environmental factors. Acting synergistically, they exert several adverse effects on health and quality of life. The longitudinal study by Kim and colleagues [32] found that an increase in visceral fat mass resulted in a decrease in skeletal muscle mass, which demonstrates the synergistic action of the two clinical entities, sarcopenia and obesity. Thus, sarcopenic obesity may have a greater effect on metabolic disorders; cardiovascular disease; increased risk of osteopenia, osteoporosis, fractures and falls; and mortality than either sarcopenia or obesity separately [33].

Sarcopenic obesity is characterized by increased fat mass with decreased muscle mass and function with a prevalence set at 20% in older adults. However, there is a lack of standard clinical diagnostic criteria for sarcopenic obesity [1,17]. Diagnostic criteria for sarcopenic obesity have not been internationally settled and various approaches are currently available with prevalence ranging from 2.75% to over 20%, depending on the criteria used. Most studies assess this health issue as a co-existence of sarcopenia and obesity. The biochemical background that leads to disability and frailty has not been defined up to now. Changes in hormonal levels may highlight the progress of scientists’ knowledge, but this field remains limited [17,18].

Changes in body composition related to aging include a gradual increase in total adipose tissue mass, re-distribution of fat tissue with preference for fat surrounding the internal organs, and reduction in subcutaneous peripheral fat. Total skeletal muscle mass reduction is up to 80% from the age of 20 years to the age of 80 years [34,35]. As mentioned above, sarcopenia is a progressive and generalized loss of skeletal muscle mass and function. The prevalence of sarcopenia was reported to be up to 29% in older persons in the community healthcare setting. Sarcopenia diagnosis is confirmed by the presence of low muscle mass plus low muscle strength or low physical performance [36]. Moreover, increased cortisol levels, insulin resistance, and decreased levels of growth hormone, insulin-like factor 1, dehydroepiandrosteron-sulfate and sex hormones, are observed, too. All these alterations affect body composition and synergistically result in the new phenotype of obesity, sarcopenic obesity [17,20,21]. Additionally, pro-inflammatory cytokine levels are rising. Both IL-6 and IL-1 are produced by hypertrophic fat cells, inter-cooperate with immune cells, and promote cascade reactions leading to myocyte apoptosis triggered by α-Tumor Necrosis Factor(α-TNF), rising levels, elevated skeletal mass, oxidative stress, and finally muscle catabolism.

Adipokines such as adiponectin and leptin have been implicated in the development of sarcopenic obesity [37]. Leptin has been correlated positively with body mass index and subcutaneous fat mass and negatively with brown adipose tissue [38] Adiponectin has been linked to sarcopenia and obesity. Sarcopenic people have been found to have increased levels of adiponectin, and adiponectin has been shown to have a protective role against breakdown of their muscle proteins [39]. Regarding the interconnection between obesity and adiponectin, obese individuals demonstrate low adiponectin levels and high insulin resistance. Obesity is related to reduced expression of adiponectin in visceral adipose tissue (VAT), and adiponectin levels have been correlated with the amount of VAT negatively, suggesting that the decrease in obesity-related adiponectin levels could potentially contribute to the detrimental impact of VAT over-accumulation on the metabolism of the whole body [40].

Leptin has been also associated with sarcopenic obesity. Serum leptin levels have been correlated with an elevated risk of sarcopenic obesity, while they are related with Skeletal Mass Index (SMI), negatively [41,42,43]. Although studies results are controversial, leptin serum levels show the amount of fat mass. Obese and sarcopenic obese olders demonstrate high circulating leptin levels due to the development of leptin resistance [38], which in turn, is caused by the decrease of the number of leptin receptors [44]. In addition, elevated leptin levels may lead to an increase in TNF-α and Il-6 which promote insulin resistance. Insulin resistance affects muscle function and quality [41], as a gradual loss of muscle strength and mass is taking place [10,11]. This chronic silent inflammatory condition is also connected to metabolic activation of chondrocytes and cells in the joints of the body [22,32].

### 2.4. Osteoarthritis

Osteoarthritis (OA), the most prevalent type of arthritis, constitutes a major cause of disability and morbidity and is an enormous burden for health and social care systems, globally affecting 528 million people [45]. The prevalence of the disease in the age group over 65 years is 40% and rates are higher in developed countries (with aging population and higher obesity rates) such as North America and Europe. The prevalence of symptomatic osteoarthritis in European countries ranges from 5.4% to 29.8% and 0.9 to 9.7% for knee and hip osteoarthritis, respectively, with some geographic heterogeneity and an association with the prevalence of obesity [45].

Osteoarthritis can affect almost all the joints of the body. Its main sites are the lower limbs, upper limbs, and spine. In the last decade, the influence of obesity on the occurrence of OA has been fully demonstrated. Indeed, obesity has been positively associated with hand OA, despite the absence of mechanical loading [46].

OA of the lower limbs is the most common joint dysfunction due to the mechanical stress they sustain. The main features of the disease include degeneration caused by destruction of articular cartilage, reduction of the inter-articular space, and development of osteophytes resulting in alteration of the joint architecture [47]. Symptoms include pain, edema, stiffness, grinding of the affected joint, limited range of motion, and, in the final stage, atrophy of muscles supporting the affected joint. Muscle atrophy resulting in loss of muscle strength, mass, and functionality (sarcopenia) appears to precede or even follow joint degeneration, obscuring the nature of this association [48]. The previous definition of OA included the term “inflammation” accompanied by redness. However, cartilage is lacking vessels and consists of chondrocytes [49]. Evidence coming from molecular biology has shown that these cells were able to take part in the homeostasis of the cartilage matrix and also were able to secrete soluble mediators known to be pro-inflammatory in other tissues, such as prostaglandins and cytokines [50].

Risk factors for the development of primary OA are age, obesity, injury and instability. Body composition assessments have shown the association of fat and lean mass with osteoarthritis, while overweight/obesity is a risk factor for knee osteoarthritis. Central obesity (measured by waist circumference and waist-to-hip ratio), is also associated with radiographic osteoarthritis of the knee [51]. Secondary OA is presented with a pre-existing joint abnormality. Predisposing factors include trauma or injury, inflammatory arthritis, hereditary joint disorders, infectious arthritis, vascular necrosis, Paget’s disease, Ehlers-Danlos syndrome osteopetrosis, osteochondritis dissecans, metabolic disorders, haemochromatosis, Wilson’s disease, and haemoglobinopathy [52].

Metabolic and endocrine disorders can also lead to the pathogenesis of OA. Evidence suggests that OA is a whole organ disease affected by systemic mediators, inflammation, immunity, and low-grade inflammation caused by the metabolic syndrome. Although all articular tissues are involved in the development of disease in OA, articular cartilage has gained the most attention in the context of aging, injury, and disease. Synovitis is a common characteristic seen in OA of all types, although there is not a clear understanding of the etiology since inflammation is heterogeneous in this disease. In addition, synovial cells express elevated levels of alarmins, such as S100A8 and -9, which have long been considered as markers of inflammation [19]. Recently, cytokines such as IL-10, IL-17, IL-22, and IL-1-Receptor Antagonist (RA) have been investigated in synovial fluid, synovial tissue, and subchondral bone from OA patients. Liquid biomarkers for prognostic use are making enormous progress with assessment in plasma and synovial fluid levels of inflammatory mediators such as IL-6, TNF-α, or IL-1 RA [24,26]. The formation of blood vessels (vascular channels) is thought to facilitate biochemical communication between bone and cartilage (such as cytokines, chemokines, leptin and alarmins), and a downward cycle of cartilage destruction begins [53].

Leptin was also detected in the synovial fluid of patients with OA, and a positive correlation has been observed between its levels in synovial fluid and the severity of OA [54]. The increased leptin levels in the osteoarthritic joint and the expression of leptin’s receptors on the surface of cartilage cells lead to the suggestion that leptin and resistin may be playing a potential role in chondral degeneration in OA and might be the key molecule that links obesity to OA. Findings coming from the clinical field have also demonstrated that leptin has a catabolic effect on articular cartilage [55,56]. In addition, resistin concentrations have been found in synovial fluid. Resistin is a polypeptide with hormonal action. In humans, resist in has the form of a dimeric protein, and its main sources are bone marrow cells, peripheral blood mononuclear cells, and macrophages, and it mediates inflammation. It is worth mentioning that increased levels of resistin in synovial fluid have been found in patients with OA and rheumatoid arthritis while elevation in serum levels is predominant [55]. The effects of reconstituted adipokine compounds on subchondral bone tissue from femoral heads of patients with hip OA who underwent total hip arthroplasty have been investigated. After stimulation of the subchondral bone from patients with normal body weight with reconstituted resistin, visfatin, or leptin, it was found that only resistin resulted in a significant rise in non-normal collagen type, which was also detected in obese patients with hip OA. In conclusion, leptin and resistin might be dominant in the metabolic type of OA [57].

Diagnosis of OA is based on imaging criteria and on the patient’s clinical status. Major symptoms are pain, stiffness, and disuse of the joint. Prolonged joint dysfunction is accompanied by a gradual muscular atrophy of the affected limb. In particular, patients with pre-existing radiographic evidence of knee and hip osteoarthritis had worse lower limb muscle strength, gait speed, and walk test, chair-rise test, and balance test scores [58,59].

Knee OA and its progressive course are statistically significantly associated with sarcopenia due to the positive effect of low muscle mass on functional lesions and to the fact that the functional status of patients with OA preoperatively is a predictor of the occurrence of complications and their recovery postoperatively [60]. Dynamopenia and sarcopenia may occur in a few weeks to two years after surgical joint reconstruction, resulting in decreased mobility, inability to perform muscle work, decreased gait speed, and disability in performing gait movement [35,39,42,47]. Nevertheless, it is worth mentioning that studies’ findings support that postoperative walking speed improved in subjects undergoing total knee arthroplasty, while the number of steps was reduced during the 20-cm step compared to those who had conservative treatment [58].

On the other hand, hip OA affects older adults, and it is a major risk for falls [27,30]. Diagnosis of clinical or self-reported knee and hip OA is connected with low physical performance affecting balance ability, gait speed, 6 min walk test, sit-to-stand and timed up and go test measurements [27,31,61]. In addition, symptoms and physical impairments that are related to lower limb OA involving joint pain and stiffness, muscle weakness, and reduced sensory function can be deleterious to balance performance and contribute to the probability of falling. Moreover, changes in joint structure, such as osteophyte formation and cartilage degeneration, may alter mobility patterns, which may in turn negatively impact balance [62,63,64,65,66]. Further, gait analysis reveals that gait differs in adults with antero-lateral and posterior exposure during hip arthroplasty compared to healthy ones [67].

### 2.5. Impact of Sarcopenia, Obesity, and Osteoarthritis on Physical Performance

It is well documented that the triad of sarcopenia, obesity, and osteoarthritis has impact on physical performance. Even middle-aged subjects with knee OA had significantly worse physical function and sarcopenic indices compared to those without OA, such as worse performance on the 40-m fast-paced walk test, lower right (*p* < 0.01) and left (*p* < 0.01) handgrip strength, sit-to-stand test, and walking speed [68]. Physical performance deficits such as lower walking speed, lower score on the sit-to-stand test, smaller range of motion, reduced grip strength and lower physical activity score have also been revealed in sarcopenic and sarcopenic obese people who underwent knee or hip arthroplasty or have knee/hip OA with mild to moderate pain preoperatively [31,35,36,69].

Regardless the co-existence of sarcopenia and/or obesity and osteoarthritis of the knee and hip, body composition alterations due to the dysfunctional structure of the affected joint are reflected in the reduced values of appendicular muscle mass index and increased values of fat mass and intramuscular fat mass [49,50]. Low bone density has also been connected to poor performance and low gait speed [70,71]. In addition, patients with hip OA who had moderate to mild pain had lower knee extension strength and less hip range of motion than the healthy ones [27,37,61,72]. Studies that reveal the association of physical performance with sarcopenia, sarcopenic obesity, and osteoarthritis of the knee and/or hip are presented in Table 2.

## 3. Conclusions

Bones, muscles, and adipose tissue are connected and interact through molecules. Mechanical loading is a fundamental mechanism that connects the three tissues with a central contributing role of physical performance. Sarcopenia and sarcopenic obesity seem to have a bidirectional relationship with the maintenance or disruption of joint architecture, highlighting an equally bidirectional cause-effect relationship by accepting the effects of ageing.

Osteoarthritis of the lower extremities, sarcopenia, and/or obesity should be managed as one entity in order to promote physical performance and quality of life and reduce the burden on the health care system.

## Figures and Tables

**Table 1:** Screening for sarcopenia.

Society/Scientific Group/	Definitions	Functional Capacity	References
ESPEN SIG (European Society on Clinician Nutrition and Metabolism special interest groups)	Muscle mass: >2SD less than mean of muscle mass in adults aged 18–39 years in 3ηNational Health and Nutrition Examination Survey	Functional capacity: Gait speed < 0.8 m/s	[10]
International Sarcopenia Initiative	As per IWGS and EWGSOP definitions Formed by international experts from the EWGSOP Formed and IWGS	Gait speed and grip strength should be used to determine low levels of muscle strength and physical performance when diagnosing sarcopenia.	[11]
EWGSOP 2	Muscle Mass: Skeletal Muscle Index (SMI) by dual X-ray absorptiometry (DEXA)]: Men < 7 kg/m^2^, Women < 5.5 kg/m^2^	Hand grip strength: Men < 27 kg, women < 16 kg Functional capacity: gait speed < 0.8 m/s Sit-to-stand test > 15 s for 5 repetitions	[12]
FNIH	Muscle Mass: Μυϊκή Muscle Mass:/body mass index (BMI) Men ≤ 0.789, women ≤ 0.512	Hand grip strength: men < 26 kg, women < 16 kg Functional capacity: gait speed < 0.8 m/s	[13]
IWGS	“Low whole-body or appendicular fat-free mass (measured using DXA) in combination with poor physical functioning”	Gait speed < 0.8 m/s	[14]
Asian Working Group for Sarcopenia AWGS	Similar to the EWGSOP working definition, but using cut-off values specific to older adults from South-East Asia	“Low muscle mass plus low muscle strength and/or low physical performance”	[15]

EWGSOP2: European Working Group on Sarcopenia in Older People; AWGS: Asian Working Group for Sarcopenia; IWGS: International Working Group on Sarcopenia; FNIH: Foundation of the National Institute of Health; ESPEN SIG: European Society on Clinician Nutrition and Metabolism Special Interest Groups.

**Table 2:** Association of physical performance with osteoarthritis, sarcopenia, and sarcopenic obesity. Studies show how bone, muscle, fat, and aging talk to each other.

	Study Design	Study Participants (N)	Physical Performance Evaluation Tools	Results	References
Knee OA	Observational Australia	N = 117 community- based population with symptomatic knee OA, N1 = 68 women N2 = 49 men Mean age 63 ± 6.2 years	MRI of knee at baseline, at 2 and 4 years Physical activity questionnaire score WOMAC score	↓ Vastus medialis (CSA) lead to ↑ Current pain, and risk knee replacement over 4 years ↑ Physical activity score negative association with WOMAC score and positive with vastus medialis CSA ↓ In vastus medialis CSA from baseline to 2-year follow up medial tibial ↑ Cartilage volume loss from 2-year follow up to 4.5-year follow up	[73]
Knee/Hip OA	Cohort	N = 2942 men and women community- based population knee/hip OA age range was 65–85 years	WOMAC Walking speed at 3 m Chair stands LASA: physical activity questionnaire (LAPAQ)	Pain without self-reported OA was present in the knee in 410 and hip in 378 individuals. ↓ Participants who had self-reported OA or clinical OA in any joint assessed: poor physical performance Stiffness in the hip and knee also showed significant positive relationships with low physical performance	[58]
Knee OA	Cross-sectional Cohort Canada	125 women: N1 = radiographic Knee OA N2 = non radiographic OA Mean age: 63.0 ± 7.2 years	Knee extensor strength: maximum isometric knee extensor strength of the right leg against a fixed force transducer Sit-to-stand test, 5 repetitions MRI scans of the thigh: Intermuscular fat Quadriceps muscle: measured by imaging software	No relationship between quadriceps muscle volume and sit to stand test. Negative relationship (r = −0.33, *p* = 0.001), between IMF and maximal isometric knee extensor strength	[74]
Hip OA	RCT (part of a Cohort Study)	N = 70 patients with hip osteoarthritis (OA) undergoing physiotherapy treatment, usual care, physiotherapy, and physiotherapy and exercise treatment Mean age: 66.5 ± 9.4 years.	TUG 40-m self-paced walk test (40-m SPWT), 30-s chair stand (30 CST), 20-cm step test in patients with hip osteoarthritis (OA) undergoing physiotherapy treatment. GRCS	N1: 84% classified as unimportant change, N2: 14% classified as major improvement group Unimportant change group statistically significant (*p* = 0.04) ↓ The number of steps performed during the 20-cm step test	[64]
Sarcopenia/ Sarcopenic obesity	Cohort	N1 = 2629 elderly subjects Mean age: 74.6 ± 6.3 years N2 = 998 young adults, (reference group) Mean age: 23.1 ± 2.8 years 4 subgroups: sarcopenic only, sarcopenic obese, obese only,	Body Composition Analysis by BIA: ASMI and SMI calculated Gait speed, TUG Handgrip Strength: handheld dynamometer	N1: ↓ ASMI, SMI and ↑ Body fat% compared to N2 Sarcopenic obesity prevalence: 25% Sarcopenic group and sarcopenic obese ↓ handgrip strength compared to N2 (*p* < 0.05) Sarcopenic only, obese only and sarcopenic obese: ↓ TUG compared to N2 (*p* < 0.05) Sarcopenic obese: ↓ TUG, gait speed, hand grip strength (*p* < 0.05) compared to N2.	[65,75]
Sarcopenic obesity and knee OA	Cohort	N = 208 subjects underwent knee arthroplasty (unilateral or bilateral knee OA) Age range: a 0.40–64 years and b. ≥ 64 years Mean age: 65.1 ± 7.9 years, Mean BMI: 37.1 ± 5.5 kg/m^2^	Sarcopenia: EWGSOP, EWGSOP 2 criteria Body Composition: DXA 6 MWT Gait speed Isometric handgrip strength: Jamar handgrip dynamometer DXA: ASM LMM calculated by ASM WC measurement	WC range: 98.8 to 158.0 cm in females; 107.7 to 162.6 cm in males Prevalence of SO: low ASM alone showed a prevalence ranging from 1.3% to 27.2%. SO: ASM by BMI: 15% in a age group, 24% in b age group (*p* < 0.05) ASM relative to body weight and BMI showed stronger positive associations with Gait speed, 6MWT, hand grip strength (*p* < 0.05) ASM with low strength or low function resulted in a prevalence of 8.6% ↑ Mobility aids in sarcopenic obesity group	[76]
Knee OA	Cross Sectional	N = 185 subjects Range of age: 40–79 years L group: at risk of locomotive syndrome Non L group: no at risk of locomotive syndrome	Body composition: BIA 6 m gait speed Stand up test Bone density: by ultrasound technique Anterior-posterior X-rays of bilateral knees for OA: Kellgrene Lawrence grade (K-L grade) knee pain assessed by JKOM score	27.0% were classified into the L group. ↓ L group compared to non-L group: bone density (*p* < 0.001), gait speed (*p* < 0.001), osteoporosis (*p* < 0.001), slow gait speed group (SGSG) (cut off 1 m/s) and JKOM score. After adjustment for age, height, weight, and gender a significant association between the stand-up test and bone density (OR 0.960, 95% confidence interval, gait speed (m/s) (OR 0.073, 95% CI), osteoporosis (OR 3.710, 95% CI), SGSG (OR 7.849, 95% CI), revealed	[77]
Sarcopenia and OA	Prospective Cohort	N = 58 subjects with end stage knee OA, underwent total knee replacement 79.3% were female 32.8% sarcopenic on baseline Mean age: sarcopenic 67.89 ± 7.07 years non sarcopenic 67.92 ± 6.85 years	WOMAC: Pain, stiffness assessment Sarcopenia assessment: Asian Working Group for Sarcopenia Body composition: by DXA Hand grip strength: Handle dynamometer Optimal isometric force of the knee flexion/ extension movement was measured by a dynamometer attached at the malleoli level. Physical activity level: IPAQ 6 m Gait speed	Sarcopenic subjects: ↓ Appendage lean mass index and lean mass lndex than non-sarcopenic ones at baseline: LMI: 13.10 (sarcopenia = yes) vs. 14.96 (sarcopenia = no); *p* < 0.01; ([ALMI] at baseline: 5.26 (sarcopenia = yes) vs. 6.11 (sarcopenia = no); *p* < 0.01 After Total Knee arthroplasty: ↓ Appendage lean mass index and lean mass index than non-sarcopenic ones after 12 months (13.39 vs. 15.42; *p* < 0.01) Hand grip strength: no change ↑ isometric force of the knee flexion/ extension movement in sarcopenic and non-sarcopenic subjects after total knee replacement ↑ Physical activity level after surgery. ↓ Gait speed 12 months after baseline; 10.24 ± 5.35 vs. 7.69 ± 2.68 (*p* < 0.01)	[78]

WOMAC: Western Ontario and McMaster Universities Osteoarthritis Index; CSA: cross-sectional area; DXA: dual-energy X-ray absorptiometry; 6MWT: 6-min walk test; GRCS: global rating of change score; SPWT: self-paced walk test; IMF: intermuscular fat volume; QM: quadriceps muscle; TUG: timed up and go; BIA: bio-impedance analysis; SMI: skeletal muscle mass index; ASMI: appendicular skeletal mass index; BMI: body mass index; OA: osteoarthritis; LMM: low muscle mass; JKOM: Japanese knee osteoarthritis measure; SLGSG: slow gait speed group; ALMI: appendage lean mass index; LMI: lean mass Index, IPAQ: International Physical Activity Questionnaire. ↑: decrease; ↓: increase.

## Data Availability

Not applicable.
